# Coix Seed Extract Enhances the Anti-Pancreatic Cancer Efficacy of Gemcitabine through Regulating ABCB1- and ABCG2-Mediated Drug Efflux: A Bioluminescent Pharmacokinetic and Pharmacodynamic Study

**DOI:** 10.3390/ijms20215250

**Published:** 2019-10-23

**Authors:** Yifan Qian, Yang Xiong, Di Feng, Yali Wu, Xu Zhang, Liping Chen, Mancang Gu

**Affiliations:** College of Pharmaceutical Science, Zhejiang Chinese Medical University, Hangzhou 311402, Zhejiang, China; qianyifan@zcmu.edu.cn (Y.Q.); xiongyang@zcmu.edu.cn (Y.X.); fengdi@zcmu.edu.cn (D.F.); wuyali@zcmu.edu.cn (Y.W.); zhangxu@zcmu.edu.cn (X.Z.); LipingChen@zcmu.edu.cn (L.C.)

**Keywords:** coix seed extract, ATP-binding cassette (ABC) transporters, gemcitabine, bioluminescent pharmacokinetic profile, D-luciferin potassium salt, pancreatic cancer

## Abstract

A deep insight into the function and kinetics of ATP-binding cassette (ABC) transporters may aid in the development of pharmaceutics that can minimize the particular facet of chemo-resistance. We utilized bioluminescence imaging to monitor the ABC transporter mediated intracellular drug efflux function. We also investigated the potential association between the intracellular bioluminescent pharmacokinetic profiles and the anti-tumor efficacy of the coix seed extract and gemcitabine against pancreatic cancer cells *in vitro* and *in vivo*. The bioluminescent pharmacokinetic parameters and pharmacodynamic index (IC_50_ and TGI) were determined. The expression levels ABCB1 and ABCG2 were assessed. Results showed that coix seed extract could synergistically enhance the anti-cancer efficacy of gemcitabine (*p* < 0.05). Meanwhile coix seed extract alone or in combination with gemcitabine could significantly increase the AUC_luc_ while decreasing the K_luc_ (*p* < 0.01). Western blot and immunohistochemistry assay demonstrated that coix seed extract could significantly mitigate gemcitabine-induced upregulation of ABCB1 and ABCG2 protein. The Pearson correlation analysis demonstrated that the bioluminescent pharmacokinetic parameters and pharmacodynamic index have strong association *in vitro* and *in vivo*. In conclusion coix seed extract could augment the efficacy of gemcitabine therapy in pancreatic cancer cells may at least partly due to the alteration of ABC transporter-mediated drug efflux function.

## 1. Introduction

Pancreatic cancer (PC) is an infamous diagnosis known for its poor prognosis, with a less than 6% overall five-year survival rate [1]. Currently, gemcitabine remains the most widely used chemotherapeutic drug in advanced pancreatic cancer [2]. However, the emergence of resistance limits the therapeutic effect of gemcitabine, leading to shortened patient survival rates [3]. There are various mechanisms of drug resistance, including drug efflux, changes in genes or signaling pathways and the influence of tumor microenvironment [4,5]. Amongst these mechanisms, ATP-binding cassette (ABC) transports mediated drug efflux is considered as one of the most critical mechanisms associated with gemcitabine resistance in pancreatic cancer [6,7,8]. 

An increasing number of traditional Chinese medicines have been widely used in combination with chemotherapeutic agents to overcome chemo-resistance [9]. Coix seed extract, which is prepared from the *Coix lacryma-jobi* (Yiyi ren) seed has been successful used to treat over 500,000 patients in China for various types of cancer, including lung cancer, pancreatic cancer and liver cancer. In 2014, the phase II clinical trial of coix seed extract for advanced pancreatic patient was also accomplished in US. Clinical studies revealed that coix seed extract can overcome chemoresistance, increase the tolerance of patients and significantly improve the life span and quality of life of patients, when combined with gemcitabine [10]. Zhou et al. [11] demonstrated that the expression of ABCG2 contributed to gemcitabine resistance in pancreatic cancer. Meanwhile, ABCB1 and ABCG2 were found to be an important indicator of drug-resistance in pancreatic cancer [12]. However, it remains largely unclear whether the coix seed extract overcome gemcitabine resistance in pancreatic cancer cells through modulating the ABC transporter activity.

Bioluminescence imaging (BLI) has become a widely utilized tool for studying biological processes in living animals [13,14,15]. Sim H and his colleagues utilized D-luciferin to monitor the tumor uptake by using BLI method [16]. Previous studies have revealed that BLI could be an appropriate approach for real-time evaluation of the intracellular efflux function of ABC transports, particularly for the efflux of chemotherapeutic agents in cancer cells *in vitro* [17,18]. However, the potential associations between the ABC transporters mediated drug efflux kinetic and efficacy of chemotherapeutic agents are largely unclear.

Following previous bioluminescent pharmacokinetic study on pancreatic cells [17] and cerebral tissue [12,17,19,20], we utilized BLI approach herein to examine whether gemcitabine and coix seed extract co-treatment could modulate the intracellular bioluminescent pharmacokinetic profiles of D-luciferin, an efflux substrate by ABCG2 and ABCB1 protein. The anti-cancer efficacy and bioluminescent pharmacokinetic parameters after exposure to the coix seed extract and gemcitabine both *in vitro* and *in vivo* was also observed to construct a kinetic model. Then the potential association between the ABC transporters mediated drug efflux activity and pharmacodynamics of gemcitabine and coix seed extract was investigated. Meanwhile, the protein expression level of ABC transporters ABCG2 and ABCB1 was examined by western blot and immunohistochemistry *in vitro* and *in vivo*.

## 2. Results

### 2.1. Determination of the Linear Correlation Range of Bioluminescence Intensity In Vitro and In Vivo

The correlation of D-luciferin concentration versus BLI signal intensity was determined. For *in vitro* study, to avoid a super-saturation effect of bioluminescence signals in pancreatic cancer cells, the D-luciferin concentration was set at a range from 0 to 8 μg/mL, which was covered within a linear correlation to the photon number of the bioluminescence signal (y = 239376x + 267912, R^2^ = 0.95), as illustrated in Supplementary Figure S1A. The cell number was also linearly related to the signal intensity (y= 455.89x − 169100, R^2^ = 0.98). As a result, 5 μg/mL D-luciferin was set as the optimal dose and 5000 cells were set as the optimal number of cells seeded in every well (Supplementary Figure S1B). As shown in Supplementary Figure S1C, the corresponding photon number exhibited a similarly increasing trend as the D-luciferin IV dosage from 50 to 150 mg/kg. Optimally, 75 mg/kg of D-luciferin was selected for the IV administration concentration. In all studies mentioned herein, every photon signaling intensity assessed after drug treatments were within a linear range and can accurately reveal the concentration of intracellular D-luciferin.

### 2.2. Coix Seed Extract Sensitized Pancreatic Cancer Cells BxPC3^luc^ and PANC-1 to Gemcitabine Exposure

**Figure 1:**
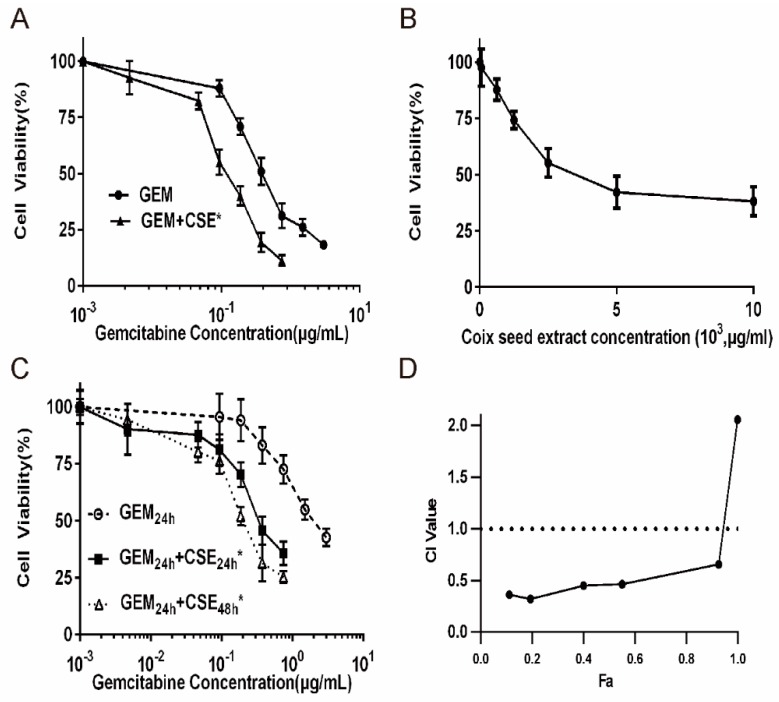
The combination of coix seed extract and gemcitabine exhibits synergistic cytotoxicity potency in pancreatic cancer cell line BxPC3^luc^. (**A**) Cell viability of BxPC3^luc^ cells exposed to gemcitabine (GEM) alone for 48 hours (IC_50_ values of gemcitabine = 0.54 ± 0.032 μg/mL) or coix seed extract (CSE) alone for 72 hours, or co-treatment of coix seed extract and gemcitabine (GEM + CSE) for 72 hours (IC_50_ values of gemcitabine = 0.13 ± 0.011 μg/mL, *p* < 0.05). *n* = 6, mean ± SD, * *p* < 0.05 vs. GEM Group. (**B**) Cell viability of BxPC3^luc^ cells exposed to coix seed extract (CSE) alone for 72 hours. *n* = 6, mean ± SD. (**C**) Cell viability of BxPC3^luc^ cells exposed to gemcitabine (GEM) alone for 24 hours (IC_50_ values of gemcitabine = 1.72 ± 0.024 μg/mL), co-treatment with coix seed extract for 24 hours (GEM_24h_ +CSE_24h_, IC_50_ values of gemcitabine = 0.30 ± 0.012 μg/mL, *p* < 0.05) or 48 hours (GEM_48h_ +CSE_48h_, IC_50_ values of gemcitabine = 0.20 ± 0.014 μg/mL, *p* < 0.05). *n* = 6, mean ± SD. * *p* < 0.05 vs. GEM Group. (**D**) The combination index (CI) of the anti-proliferation effects of gemcitabine (GEM) and coix seeds extract (CSE) in BxPC3^luc^ cells. Fa refers to cell viability inhibition rate. CI > 1 represents antagonistic cytotoxicity; CI = 1 represents addictive cytotoxicity; CI < 1 represents synergistic cytotoxicity.

The 3-(4, 5-dimethylthiazolyl-2)-2, 5-diphenyltetrazolium bromide (MTT) assay was used to determine whether coix seed extract can enhance the cytotoxicity of gemcitabine in BxPC3^luc^ and PANC-1 cell lines. And coix seed extract (10 mg/mL) and gemcitabine (3 μg/mL) were chosen for further *in vitro* studies. We found that the co-treatment of two drugs was more effective in sensitizing the BxPC3^luc^ and PANC-1 cells to gemcitabine exposure (Figure 1, Supplementary Figure S7). Results showed that co-treatment with coix seed extract significantly decreased the IC_50_ of gemcitabine from 0.54 to 0.13μg/mL (Figure 1A). To further confirm the synergism between the coix seed extract and gemcitabine, BxPC3^luc^ and PANC-1 cells were treated with gemcitabine and coix seed extract for 24 and 48 hours. It was shown that treatment with the coix seed extract treatment for either 24 or 48 hours could lower the gemcitabine IC_50_ from 0.30 to 0.20 μg/mL (BxPC3^luc^ cells, Figure 1C) and 0.35 to 0.29 μg/mL (PANC-1 cells, Supplementary Figure S7A), while the IC_50_ of gemcitabine alone was 1.72 μg/mL (Figure 1C). In PANC-1 cell model, co-treatment with coix seed extract could significantly decrease the IC_50_ of gemcitabine (1.1-fold to 1.3-fold, *p* < 0.01) after 24 to 48 hours treatment (Supplementary Figure S7A). CI values for the combination treatment group were also calculated. CI refers to combination index; where CI < 1 represents synergism, CI >1 represents antagonism and CI = 1 represents an additive effect. As shown in Figure 1D, BxPC3^luc^ treated with gemcitabine showed synergistic loss of cell viability when the gemcitabine was combined with the coix seed extract (combination index = 0.54). And a similar CI value (combination index = 0.67) was observed in PANC-1 cell line treated with coix seed extract and gemcitabine (Supplementary Figure S7B).

### 2.3. The Coix Seed Extract Significantly Reversed Gemcitabine-Induced Decreases in Intracellular Bioluminescent Signal and Gemcitabine Accumulationin BxPC3^luc^ Cells

**Figure 2:**
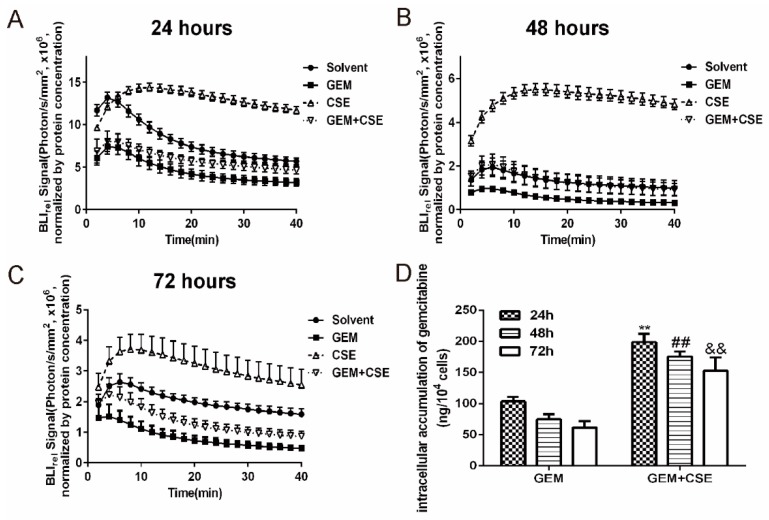
Relative bioluminescence imaging (BLI_rel_) signal-time curves of D-luciferin and intracellular accumulation of gemcitabine in BxPC3^luc^ cells.Cells were first exposed to gemcitabine (GEM, 3 µg/mL), coix seed extract (CSE, 10 mg/mL) or the combination of gemcitabine and coix seed extract (GEM, 3 µg/mL; CSE, 10 mg/mL) for 24 hours (**A**), 48 hours (**B**) and 72 hours (**C**). Then each group of cells was treated with 5 µg/mL D-luciferin. The bioluminescent signal of each group was captured immediately for 40 minutes in the IVIS imaging system. BxPC3^luc^ cells treated with dimethyl sulfoxide (DMSO) represented a control group. The bioluminescent intensity of each group was normalized by protein concentration and regarded as relative bioluminescent intensity (BLI_rel_). *n* = 6, mean ± SD. (**D**) The intracellular amounts of gemcitabine after different drug exposure for the indicated time were determined by liquid chromatography mass spectrometry. ** *p* < 0.01 vs. GEM_24h_, ## *p* < 0.01 vs. GEM_48h_, &&*p* < 0.01 vs. GEM_72h_. *n* = 6, mean ± SD.

Gemcitabine is reported to induce multidrug resistance (MDR) in pancreatic cancer cells [21]. While the underlying mechanisms remain unclear, the upregulation of intracellular drug efflux and increased drug elimination rate induced by gemcitabine are regarded as one of the most important mechanisms. The enhanced cytotoxicity shown by the combination treatment is hypothesized to be a product of the coix seed extract modifying gemcitabine drug efflux in pancreatic cancer cells BxPC3^luc^. D-luciferin was used as an intracellular bioluminescent probe, which is a substrate of ABC transporters [19,22]. The intracellular bioluminescent pharmacokinetic profiles were measured in BxPC3^luc^ cells. As shown in Figure 2, the BLI_rel_ versus time curves were plotted for each treatment group. Gemcitabine treatment limited the intracellular bioluminescent signals intensity compared with DMSO (*p* < 0.01). However, the presence of the coix seed extract not only induced higher bioluminescent signals than that of DMSO but also reversed the gemcitabine-induced decreases in bioluminescent signal in BxPC3^luc^ cells.

Thereafter the BLI_rel_ was calculated as described above [17] and the pharmacokinetic parameters AUC_luc_, K_luc_, and MRT_luc_ were calculated using DAS 4.0 pharmacokinetic analysis software (Anhui, China) according to the non-compartment model (Table 1). Our study showed that compared with DMSO, the AUC_luc_ and MRT_luc_ of D-luciferin significantly decreased in a time-depended manner after gemcitabine treatment in BxPC3^luc^ pancreatic cells, while the K_luc_ significantly increased when compared with DMSO. This result indicated that the intracellular bioluminescent substrate was significantly accumulated when cells exposed to gemcitabine, which was consistent with our previous study [17]. Meanwhile, coix seed extract and gemcitabine co-treatment could increase the AUC_luc_ (1.3-fold to 1.7-fold, *p* < 0.01) after 24 to 72 hours treatment comparing with gemcitabine alone group. The combination group also revered the increasing trend of K_luc_ after exposing to gemcitabine alone. Because the AUC_luc_ and K_luc_ represent the intracellular accumulation and the elimination of D-luciferin, our data showed that coix seed extact could rescue gemcitabine induced the decline of intracellular accumulation of D-luciferin, which results in a higher bioluminescent signal intensity. To further confirm our bioluminescent pharmacokinetic results, we performed the ultra-high performance liquid chromatography-mass spectrometry to determine the intracellular accumulation of gemcitabine in BxPC3^luc^ pancreatic cells. As shown in Figure 2D, the accumulation of gemcitabine decreased in a time-dependent manner when gemcitabine was used alone. Meanwhile co-treatment with coix seed extract could significantly increase the intracellular accumulation of gemcitabine in both BxPC3^luc^ and PANC-1 cells (Figure 2D, Supplementary Figure S7C). Most notably, intracellular gemcitabine significantly increased (*p* < 0.05) when cells were incubated alongside the ABCG2 inhibitor Ko143 or ABCB1 inhibitor verapamil, which indicating that inhibition of function of ABCG2 and ABCB1could reverse decrease in intracellular gemcitabine accumulation in both BxPC3^luc^ and PANC-1 cells (Figure S8). These data were consistent with the trend of AUC_luc_ while receiving the same exposure and indicated that the coix seed extract can significantly reverse the gemcitabine induced intracellular drug efflux and elimination while increasing intracellular drug accumulation in BxPC3^luc^ cells.

### 2.4. Coix Seed Extract and Gemcitabine in Combination Exhibited Potential Antitumor Growth Efficacy

**Figure 3:**
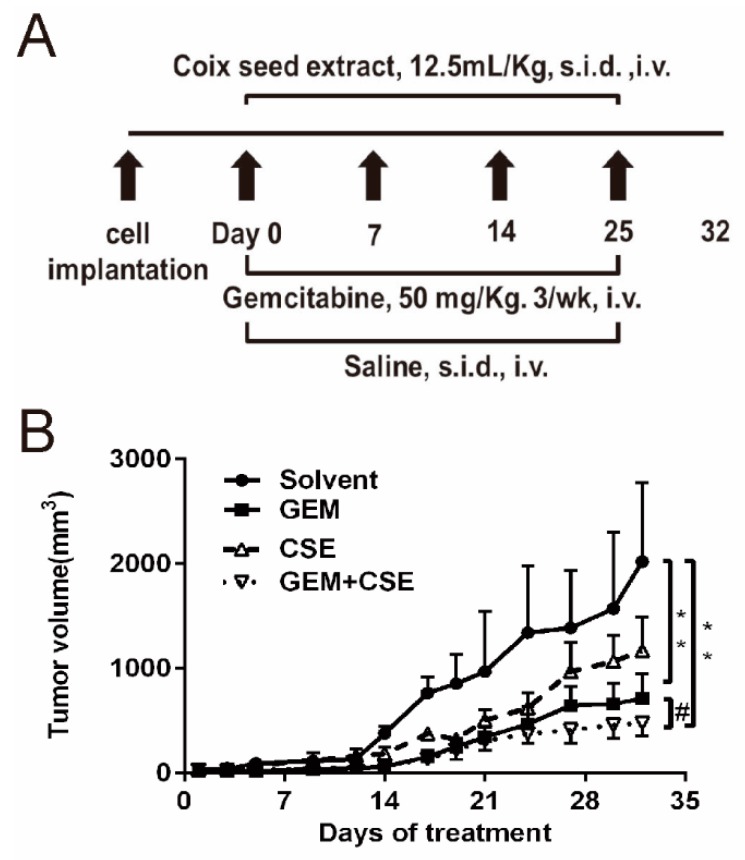
Coix seed extract enhances the antitumor effect of gemcitabine in xenograft nude mice implanted with BxPC3^luc^ cells. (**A**) Brief presentation of *in vivo* experimental protocol. (**B**) Average tumor volume of solvent and different drug treatment groups was determined every three days after the first administration until day 32. *n* = 6, mean ± SD. ** *p* < 0.01 vs. solvent group; # *p* < 0.05 vs. gemcitabine alone group.

Based on our *in vitro* findings that co-treatment of coix seed extract and gemcitabine sensitizes gemcitabine-induced cytotoxicity in BxPC3^luc^ cells, the therapeutic efficacy of the coix seed extract and gemcitabine was then evaluated in a xenograft nude mouse model implanted with BxPC3^luc^ cells. Compared with the untreated solvent group, the coix seed extract could reduce the tumor volume, while gemcitabine treatment alone caused a significant reduction in tumor volume (*p* < 0.05). It was found that gemcitabine combined with the coix seed extract showed the highest efficacy among all groups (*p* < 0.01) (Figure 3B). Meanwhile, the coix seed extract partly reversed the severe weight loss in the mice induced by gemcitabine indicating that the coix seed extract may reduce the gemcitabine caused toxicity to mice (Supplementary Figure S3). The gemcitabine and coix seed extract in combination had greatest tumor inhibition rate of 76.01 ± 8.46% (*p* < 0.05) while the tumor inhibition rates of the coix seed extract and gemcitabine alone are 44.52 ± 4.68% and 67.85 ± 12.77%, respectively. Therefore, the co-treatment group demonstrated far greater anti-tumor efficacy than that of the coix seed extract (*p* < 0.05) or gemcitabine used alone.

### 2.5. The Characterization of the Intracellular Pharmacokinetic Profiles of Bioluminescence In Vivo after Exposure to Coix seed Extract and Gemcitabine

**Figure 4:**
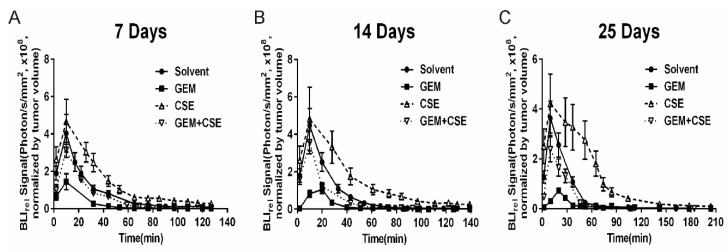
Relative bioluminescence imaging (BLI_rel_) signal-time curves of D-lucferin in xenograft nude mice implanted with BxPC3^luc^ cells. Xenograft nude mice were treated with gemcitabine (GEM, 50 mg/Kg, 3/wk, i.v.), coix seed extract (CSE, 12.5 mL/Kg, q.d., i.v.) or the combination of gemcitabine and coix seed extract (GEM, 50 mg/Kg, 3/wk, i.v.; CSE, 12.5 mL/Kg, q.d., i.v.). At days (**A**) 7, (**B**) 14 and (**C**) 25 after the first administration of treatment, all mice were weighed and anaesthetized by isoflurane gas and injected with 75 mg/kg of D-Luciferin solution intravenously. The bioluminescent signal of each group was captured immediately for 140 minutes in the IVIS imaging system. BxPC3^luc^ cells treated with saline (q.d., i.v.) represented as control group. The bioluminescent signals intensity of each mouse was normalized by tumor volume and considered as BLI_rel_. *n* = 6, mean ± SD.

In our *in-vitro* bioluminescent assay, the gemcitabine and coix seed extract combination treatment increased the AUC_luc_ and decreased the K_luc_ compared with gemcitabine treatment alone. To further explore the intracellular bioluminescent pharmacokinetic profiles after exposure to coix seed extract and gemcitabine, we employed a xenograft nude mouse model bearing BxPC3^luc^ cells (see experimental theme in Figure S3A). At the day 7, 14 and 25 post the first drug administration, all xenograft mice were injected 75 mg/kg of D-luciferin solution intravenously and were immediately subjected to bioluminescence imaging for 140 minutes. The bioluminescent pharmacokinetic profiles of animals were shown in Figure 4. The pharmacokinetic parameters were calculated according to the non-compartment model (Table 2). It was found that the AUC_luc_ (2.2-fold to 2.5-fold, *p* < 0.01) and MRT_luc_ (1.5-fold to 1.8-fold, *p* < 0.01) of the bioluminescent signal intensity of coix seed extract treatment were significantly raised among all groups at all three time points, while the gemcitabine treatment led to a significant decreasing of AUC_luc_ (4.3-fold to 5.6-fold, *p* < 0.01) and MRT_luc_ (1.2-fold to 1.5-fold, *p* < 0.01) and increasing of K_luc_ (1.3-fold to 1.6-fold, *p* < 0.01) comparing with the solvent group. In addition, the coix seed extract could significantly reverse gemcitabine induced downregulation of AUC_luc_ (2.9-fold to 3.6-fold, *p* < 0.01) and MRT_luc_ (1.1-fold to 1.6-fold, *p* < 0.01) and upregulation of K_luc_ (1.3-fold to 1.7-fold, *p* < 0.01) when combined with gemcitabine. Consistent with the *in vitro* bioluminescent pharmacokinetic findings, our *in vivo* data also indicated that coix seed extract may rescue gemcitabine mediated drug efflux activity of pancreatic cancer cells in a time dependent manner. 

### 2.6. The Intracellular Bioluminescent Pharmacokinetic Parameter AUC Showed Potential Associations with Anti-Pancreatic Cancer Efficacy In Vitro and In Vivo

**Figure 5:**
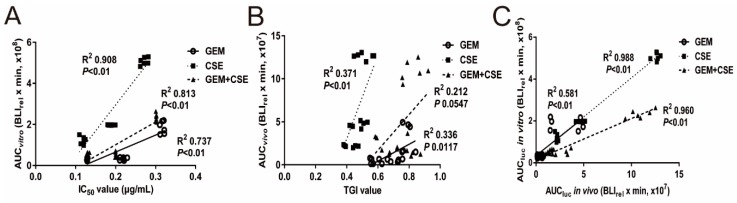
The intracellular bioluminescent pharmacokinetic parameter AUC shows a strong association with anti-tumor efficacy *in vitro* and *in vivo.* (**A**) BxPC3^luc^ cells were exposed to GEM, CSE or GEM and CSE combination for 24, 48 and 72 hours. The correlation coefficient R^2^ and *p* value between the bioluminescent pharmacokinetic parameter AUC_luc_ and the anti-proliferation parameter IC50 for each drug at each treatment time point were determined by Pearson correlation model. (**B**) The xenograft mice bearing BxPC3luc cells were intravenously injected with GEM, CSE or GEM and CSE combination. The bioluminescent pharmacokinetic parameter AUCluc and the anti-proliferation parameter TGI were measured on days 7, 14 and 25. The correlation coefficient R2 and *p* value between AUCluc and TGI for each drug and treatment time point were determined by Pearson correlation model. (**C**) The association between in vitro AUCluc and in vivo AUCluc for GEM, CSE or GEM and CSE combination at each measurement time point were compared. The correlation coefficient R2 and *p* value were determined by Pearson correlation model.

To determine the potential association between anti-cancer efficacy and the pharmacokinetic profiles of bioluminescence of different treatment groups at various time points, we utilized the Pearson correlation analysis. The IC_50_ values from *in-vitro* data and the tumor growth inhibition rate (TGI) from *in-vivo* data were selected as efficacy indexes. AUC_luc_, K_luc_ and MRT_luc_ were selected as indexes for kinetic of bioluminescent intensity. As shown in Figure 5A, the co-treatment group demonstrated a lower IC_50_ and higher AUC_luc_ than that of the gemcitabine treatment group. Interestingly, AUC_luc_ data was strongly and positively correlated with IC_50_ value, while the R-Square of relevance coefficient (R^2^) of the gemcitabine, coix seed extract, and co-treatment were 0.74 (*p* < 0.01), 0.91 (*p* < 0.01) and 0.81 (*p* < 0.01), respectively. The same associations can be found in the *in vivo* assay in Figure 5B. The *in vivo* AUC_luc_ versus tumor growth inhibition rate in each treatment group only showed moderate correlation, and the R^2^ of the gemcitabine, coix seed extract, and co-treatment were 0.34 (*p* = 0.012), 0.37 (*p* < 0.01) and 0.21 (*p* = 0.055), respectively, which might be due to the larger variance of *in vivo* data. Meanwhile, a strong positive correlation between *in vitro* AUC_luc_ and *in vivo* AUC_luc_ of each treatment group was also observed in Table 3. The correlation coefficient R^2^ of the gemcitabine, coix seed extract, and co-treatment were 0.58 (*p* < 0.01), 0.99 (*p* < 0.01) and 0.96 (*p* < 0.01) respectively, which indicated a similar variation trend of intracellular bioluminescent pharmacokinetic profiles after drug treatment between *in-vivo* and *in-vitro* data. As shown in Table 4, the *in-vitro* proliferation rate of the three groups (gemcitabine, coix seed extract, gemcitabine and coix seed extract combination) was negatively correlated with the MRT_luc_ and AUC_luc_, and a positive correlation with K_luc_. 

### 2.7. Coix Seed Extract Reverses Gemcitabine-Induced Upregulation ABCB1 and ABCG2 Protein Level In Vitro and In Vivo

**Figure 6:**
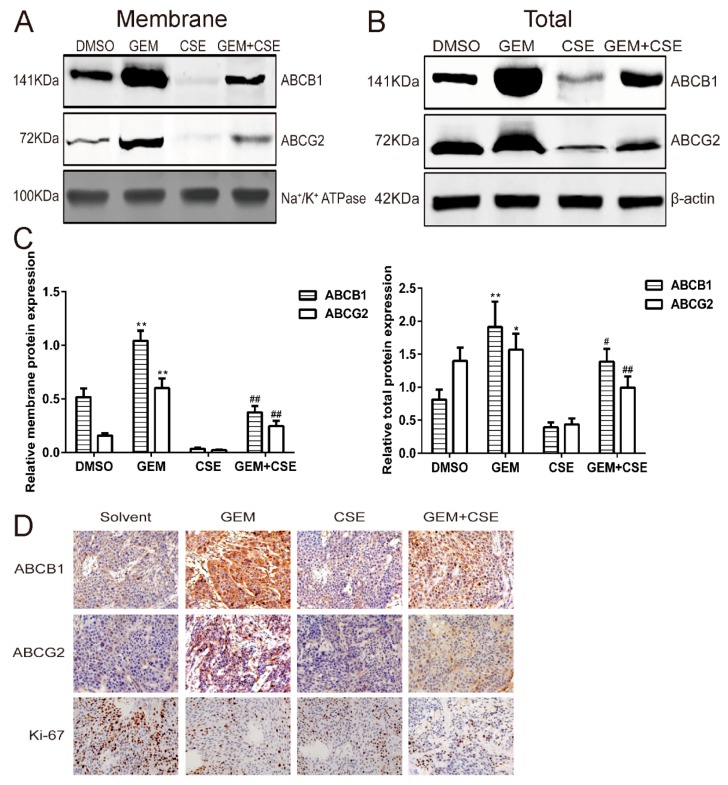
Coix seed extract reverses gemcitabine induced upregulation of ABCG2 and ABCB1 protein *in vitro* and *in vivo*. Results are representative of at least three independent experiments. (A) The coix seed extract induces downregulation of membrane ABCG2 and ABCB1 protein expression in BxPC-3^luc^ cells. Na^+^/K^+^ ATPase was utilized as loading control. (B) The coix seed extract induces downregulation of total ABCG2 and ABCB1protein expression in BxPC-3^luc^ cells. β-actin served as loading control. (C) Relative integrated optical density (IOD) of ABCG2 and ABCB1 protein expression in membrane and total protein proportion of BxPC-3^luc^ cells. *n* = 3, mean ± SD. ** *p* < 0.01 vs. DMSO; * *p* < 0.05 vs. DMSO; ## *p* < 0.01 vs. gemcitabine (GEM) treatment; # *p* < 0.05 vs. gemcitabine (GEM) treatment. (D) Immunohistochemical demonstration of ABCG2, ABCB1 (ABC transporters) and Ki67 (cell proliferation) protein expression in tumor tissue harvested from pancreatic cancer xenograft mice intravenous injected GEM, CSE or GEM and CSE combination (original magnification: ×400).

The expression and activity of ABCB1 and ABCG2 are two major biomarkers for gemcitabine-induced MDR [3,4,23]. To further explore the possible mechanism of the association between AUC_luc_ and anti-cancer efficacy for each drug treatment group both *in vitro* and *in vivo*, western blotting and immunohistochemical analysis were used to detect the ABCB1 and ABCG2 expression following drug treatments *in vitro* and *in vivo*. As shown in Figure 6A, gemcitabine treatment can upregulate ABCB1 and ABCG2 level in cell membrane protein fraction to 2.0-fold (*p* < 0.01) and 3.8-fold (*p* < 0.05) in pancreatic cancer cell BxPC3^luc^ compared with DMSO, while the coix seed extract treatment decreased ABCB1 and ABCG2 level in cell membrane protein fraction to 15.0-fold (*p* < 0.05) and 6.8-fold (*p* < 0.01). Interesting, a significant downregulation of ABCB1 and ABCG2 expression levels in cell membrane protein fraction to 2.8-fold (*p* < 0.01) and 2.4-fold (*p* < 0.01) was also found after coix seed extract and gemcitabine co-treatment when compared with gemcitabine alone treatment. The same trend of ABCB1 and ABCG2 in cell total protein fraction was observed (Figure 6B). Our findings indicated that the co-treatment with coix seed extract may reverse gemcitabine-induced upregulation of ABCB1 and ABCG2 expression.

In order to further confirm the protein expression results *in vitro*, the immunohistochemical analysis was assessed for ABCB1 and ABCG2 expression levels in pancreatic tumor tissues from xenograft mice. As shown in Figure 6D, ABCB1 and ABCG2 are positively expressed on the cell membrane showing a brown color. Gemcitabine upregulated the expression level of ABCB1 and ABCG2 in the tumor section when compared with solvent (*p* < 0.05). On the other hand, a significant downregulation of ABCB1 and ABCG2 protein was observed in tumor tissue specimens from both coix seed extract and gemcitabine co-treatment treatment group (*p* < 0.05) or coix seed extract alone treatment group (*p* < 0.05) comparing with gemcitabine alone treatment group. Meanwhile, a reduction in Ki-67 staining in tumor tissues from each drug treatment group indicated a lower rate of cell proliferation.

## 3. Discussion

The substantial correlation between expression of human ABC transporters and the critical role of multidrug resistance efflux pumps in intrinsically drug-resistant cancers such as pancreatic, colon, liver cancers led naturally to development of many inhibitors of ABC transporters. However, most clinical works were failed due to intolerable toxicity or no significant treatment response [24]. Thus, it is worth to explore new ABC transporter inhibitor with low toxicity and high efficacy from Chinese herbal medicine. Coix seed extract was reported to enhance the anti-pancreatic cancer efficacy of gemcitabine in different preclinical studies [17,25,26,27] and phase II clinical trial in US [10]. Current molecular pharmacology studies have revealed that coix seed extract may show anti-pancreatic cancer activity through multiply mechanisms including inducing apoptosis and cell cycle arrest at G2/M phase [25,28], inhibition of NF-kB pathway [29,30] and PI3K/AKT pathway [31]. However, it remains unclear whether the coix seed extract could suppress ABC transporter mediated drug efflux which was induced by gemcitabine and enhance the efficacy of chemotherapy in pancreatic cancer. In this study, a pancreatic cancer cell line expressing firefly luciferase (BxPC3^luc^) and bioluminescent imaging were employed to investigate the profiles of ABC transporter mediated drug efflux kinetic and anticancer efficacy after exposure to coix seed extract and gemcitabine *in vitro* and *in vivo*. As a result, a significant association between the bioluminescent pharmacokinetic parameters (AUC_luc_, MRT_luc_ and K_luc_) and an improvement of anti-cancer efficacy of the coix seed extract and gemcitabine co-treatment was found both in cells and the xenografts mice model. 

*In vitro* cell viability results indicated that coix seed extract can enhance the cytotoxicity of gemcitabine against BxPC3^luc^ and PANC-1 cells and showed a clear degree of synergism (Figure 1, Supplementary Figure S7). Moreover, the animal study also demonstrated the synergistic effect of coix seed extract and gemcitabine against pancreatic tumor growth (Figure 3B) which is consisted with our *in vitro* data and other previous studies [28,30,32]. Zhang et al. [32] found that the coix seed extract could prevent cisplatin resistance by inhibiting the ABCB1 and ABCC1 expression and enhance the sensitivity of cells to cisplatin in gastric cancer cells. Gemcitabine was reported to induce the activity of ABC transporters and enhance the drug efflux in cancer cells [23,33,34]. Except for ABC transporters mediated drug efflux, various possible mechanisms involved in gemcitabine mediated chemo-resistance, including the expression of NT5C1A and hCNT3, changes in the expression or function of gemcitabine metabolizing enzymes and alterations in cell proliferation, survival or apoptosis signaling pathways [23]. The expression of the main four factors (hENT1, dCK, RRM1 and RRM2) involved in gemcitabine transport and metabolism was also shown to correlate with acquired resistance to gemcitabine in pancreatic cancer cells [35]. Different cell lines may provide different aspects of chemo-resistance mechanisms. However, in our previous study [17,18] we employed different cancer cell lines including BxPC3^luc^, PANC-1^luc^ and MCF-7/DOX^luc^ as cell models and confirmed that gemcitabine and doxorubicin could upregulate the ABCG2 mediated drug efflux function in different cell lines. Moreover, our previous study and other articles showed the ABC transporters function played a key role in chemo-resistance in pancreatic cancer cell lines BxPC3 and PANC-1 [12,36]. Therefore, we next assessed whether coix seed extract could modulate the drug efflux effect regulated by the ATP binding cassette transporters ABCG2 and ABCB1 in pancreatic cancer cells *in vitro* and *in vivo*.

To date, it is still lack of appropriate approach to monitor intracellular drug efflux function in a real-time and non-invasion model of target cells in live animals [15,37,38]. Bioluminescent imaging is widely used to dynamic monitor the cell growth and location in live animals due to its high signal to noise ratio (SNR), low toxicity and high sensitivity [39]. However, recently studies demonstrate that D-luciferin potassium salt, a substrate of firefly luciferase is also a substrate of ABC transporters ABCG2 and ABCB1 [12,17,40]. When the intracellular luciferase enzyme is unsaturated, the bioluminescent intensity will follow a linear correlation with the amount of D-luciferin potassium salt compounds in cells but not the number of labeled cells [17,41]. Hence, currently bioluminescent imaging is considered as a new and powerful method to monitor intracellular drug efflux function of special type of cells in tumor and cerebral tissues [17,18,19,41]. Our previous study has shown that gemcitabine had no significant effect on firefly luciferase activity and has excluded the possibility that modulation of luciferase activity may confound BLI signal intensity [17]. Meanwhile, our group and other researchers also showed that inhibition of ABCG2 and ABCB1 activity could alter the bioluminescent signal in cells overexpressed *firefly luciferase gene* [17,40]. Therefore, D-luciferin potassium salt could be utilized as a probe for determining intracellular bioluminescent pharmacokinetics following coix seed extract and gemcitabine treatments *in vitro* and *in vivo*. 

The results of *in vitro* and *in vivo* bioluminescent assays all agreed that coix seed extract and gemcitabine co-treatment could significantly increase the key pharmacokinetic parameters AUC_luc_ and MRT_luc_ while decrease K_luc_ when compared with gemcitabine monotherapy (Figure 2 and Figure 4). The increasing trends of the AUC_luc_ and the decreasing trend of K_luc_ indicated the intracellular accumulation of D-luciferin potassium salt increased while the elimination rate decreased, which consequently resulted in a higher bioluminescent signal intensity. Our bioluminescent pharmacokinetic results were consistent with reports from other studies [16] and may indicate the alternation of ABC transporter-mediated drug efflux function in cells after drug exposure. 

To further verify that the accumulation of intracellular gemcitabine in pancreatic cancer cells was directly regulated after drug exposure, a liquid chromatography mass spectrometry assay was established as an additional measurement. The amount of gemcitabine in BxPC3^luc^ and PANC-1 cells after gemcitabine alone and combined with coix seed extract was determined in each time point. Compared with gemcitabine monotherapy, co-treatment with the coix seed extract significantly increased the intracellular accumulation of gemcitabine (Figure 2D, Supplementary Figure S7C), which was consistent with the changing trends of bioluminescent pharmacokinetic parameters, AUC_luc_, K_luc_ and MRT_luc_. Accumulation research implied that ABC transporters may play a key role on the gemcitabine resistance in pancreatic cancer [6,11,12,23]. Ko143 is a widely reported inhibitor of breast cancer resistance protein (BCRP/ABCG2) [42] and significantly enhanced the sensitivity of PANC-1-R cells to gemcitabine by increasing drug efflux [43]. And verapamil, an ABCB1 inhibitor, resensitized the resistant cells to gemcitabine in a dose-dependent manner by promoting intracellular drug accumulation to combat chemotherapeutic resistance [44,45]. So it’s essential to study the effect of ABC transporter inhibitors on the gemcitabine efflux activity. Next we investigated the potential effect of ABCG2 inhibitors (Ko143) and ABCB1 inhibitors (verapamil) on the intracellular accumulation of gemcitabine to further confirm ABC transporter function on gemcitabine efflux in pancreatic cancer cells. ABCG2 inhibitor Ko143 or ABCB1 inhibitor verapamil significantly increased the intracellular gemcitabine (*p* < 0.05), indicating that inhibition the function of ABCG2 and ABCB1 could reverse intracellular drug efflux activity in BxPC3^luc^ and PANC-1 cells (Figure 2D, Supplementary Figure S8), which was consistent with our previous study [17,18]. The intracellular gemcitabine accumulation data also implied that the enhancement of coix seed extract to the cytotoxicity of gemcitabine may associated with the increasing the availability of gemcitabine for pancreatic cancer cells [30,46].

Next we asked whether the modulation of ABC transporters mediated drug efflux function by coix seed extract and gemcitabine could have association with the anti-pancreatic cancer effect both *in vitro* and *in vivo*. To this end, we conducted a Pearson correlation analysis between pharmacodynamics parameters (IC_50_ value, TGI value) and bioluminescent pharmacokinetic parameters (K_luc_, AUC_luc_ and MRT_luc_). Our correlation data showed that AUC_luc_ had a strong association with anti-tumor efficacy for each drug treatment group both *in vitro* and *in vivo* (Figure 5A, 5B). Furthermore, the *in vitro* and *in vivo* AUC_luc_ data also showed a strong correlation with each other in different treatment groups (Figure 5C). Our correlation data indicated that the coix seed extract enhanced anti-cancer efficacy of gemcitabine in pancreatic cancer cells was strong associated with the downregulation ABC transport mediated drug efflux, which may lead to increased gemcitabine accumulation in cells. Previous studies have already indicated that pharmacokinetic indexes could reflect the process of tumorigenesis and cancer development when monitoring of therapeutic efficacy using bioluminescent imaging [18,30,47]. Although there are several pathways for regulating gemcitabine efflux and influx, ABC transporters may be essential and critical for inducing gemcitabine resistance [3]. In addition, ABCB1 and ABCG2 transporter mainly expresses in cell membranes and cytoplasm in pancreatic cancer cells [6,48]. To further confirm our findings, ABCG2 and ABCB1, two major ABC transporters of gemcitabine [11,49,50,51] and also the specific transporters for D-luciferin potassium salt [19], were assessed by western blot and immunohistochemical analysis *in vitro* and *in vivo*. Unsurprisingly, gemcitabine significantly upregulated the expression level of ABCG2 and ABCB1 in both cell membrane fraction and total protein, while the coix seed extract could reverse the gemcitabine-induced increase of ABCG2 and ABCB1 protein expression. Furthermore, the immunohistochemical staining results of ABCG2 and ABCB1 also confirmed the *in vitro* data. Specifically, ABCG2 and ABCB1-medicated efflux could lead to gemcitabine resistance [7,23]. Taking together, our intracellular bioluminescent pharmacokinetic data and the expression of ABCG2 and ABCB1 both suggest that the improvement of anti-pancreatic cancer efficacy when combined gemcitabine with coix seed extract may at least partly due to that coix seed extract could reverse gemcitabine induce mediated drug efflux function mediated ABCG2 and ABCB1. 

Even the bioluminescent imaging we conducted in the present study is a real-time and non-invasion approach to determine the intracellular drug efflux function of target cells *in vitro* and *in vivo*. Our current study still has some limitations. We could only monitor intracellular bioluminescent pharmacokinetic profiles in cells transfected with firefly luciferase but not cells in whole animal bodies. The bioluminescent signal intensity could linearly correlate with the amount of intracellular D-luciferin potassium salt only within a relatively narrow range of D-luciferin potassium salt concentration. That obviously limited the time range of observation which is usually no more than three hours. Moreover, even one photon of bioluminescent signal is theoretically reflected one molecule of D-luciferin potassium salt, the bioluminescent imaging approach still cannot provide the intracellular concentration of D-luciferin potassium salt as accurate as high-performance liquid chromatography (HPLC) or mass spectrometry (MS). Moreover, even we have conducted our research in two pancreatic cancer cell lines, we will test the treatment response of coix seed extract and gemcitabine in a larger pancreatic cancer cell panels since the ABC transporters-based drug efflux in chemo-resistance may not be significant to all pancreatic cancers. Our current data and previous study [17] both showed that the ABCG2 and ABCB1 inhibitor could also regulate the intracellular bioluminescent signal intensity with or without the present of gemcitabine, we are going to employ some classical ABC transporter inhibitors and CRISPR mediated knockout approaches to further investigate the molecular functions that ABC transporters might play in the synergistic effect of coix seed extract and gemcitabine combination against pancreatic cancer cells. Despite the drawbacks above, bioluminescence imaging is still a new and powerful approach to assess the ABC transporters regulated intracellular drug efflux function of cancer cells both *in vitro* and *in vivo*.

In summary, a bioluminescence imaging method was used herein to investigate the potential mechanisms by which the coix seed extract greatly enhanced anticancer efficacy when combined with gemcitabine. Particularly, the kinetic profiles of ABC transporter mediated drug efflux and drug efficacy after coix seed extract and gemcitabine treatment both *in vitro* and *in vivo* were observed. It was found that the coix seed extract sensitized BxPC3^luc^ cells to gemcitabine exposure and could reverse gemcitabine-induced resistance, which may be due to downregulation of ABC transporter mediated drug efflux in pancreatic cancer cells. A clear and better understanding of the potential mechanisms behind gemcitabine-mediated drug resistance serves to reduce the potential for systemic side effects while improving the anticancer efficacy of similar therapeutic strategies.

## 4. Materials and Methods

### 4.1. Materials

D-luciferin was purchased from Science light Biology Science & Technology Co. Ltd (Shanghai, China). 2’-deoxy-2’,2’-difluro cytidine (gemcitabine, purity degree > 99%) was purchased from the National Institute for Food and Drug Control (Beijing, China). The coix seed extract (CSE) was an injectable emulsion form of coix seed oil which prepared by Zhejiang Kanglaite Pharmaceutical Co., Ltd, using pharmaceutical grade coix lachryma-jobi seeds. The main active ingredients of CSE are linolein, dilinoleic acid-triolein, palmic acid-dilinolein, linoleic acid-glyceryl dioleate, palmic acid-linoleic acid-triolein, glyceryl trioleate and palmic acid-glyceryl dioleate [32,52]. RPMI1640 medium and fetal bovine serum (FBS) were purchased from Gibco (Invitrogen Inc., Carlsbad, CA, USA). Trypsin–EDTA, PBS, penicillin, dimethyl sulfoxide (DMSO), streptomycin and 3-(4,5-Dimethyl-2-thiazolyl)-2, 5-diphenyl-2H-tetrazolium bromide (MTT) were all purchased from Sigma (St. Louis, MO, USA). The BCA Protein Assay Kit was from Beyotime Institute of Biotechnology (Shanghai, China). 

### 4.2. Cell Culture

Pancreatic cancer cell lines expressing luciferase (BxPC3^luc^ cells) were obtained from the Science light Biology Science & Technology Co. Ltd (Shanghai, China). PANC-1 cells were obtained from the the Type Culture Collection of the Chinese Academy of Sciences (Shanghai, China). Cells were cultured in RPMI1640 medium containing 10% FBS, 100 IU/mL penicillin, and 100 mg/mL streptomycin at 37 °C with 5% CO_2_ in a humidified atmosphere. Culture medium was changed every 2 days. All experiments were performed on cells in the logarithmic growth phase.

### 4.3. Cell Viability Assay

BxPC3^luc^ and PANC-1 cells were placed into 96-well plates at a final concentration of 5 × 10^3^ cells/well in complete culture medium and allowed to attach for 24 h. Subsequently the cells were incubated with the coix seed extract (10, 5, 2.5, 1.25, 0.63 and 0.063 mg/mL), gemcitabine (3, 1.5, 0.75, 0.37, 0.19 and 0.094 µg/mL) alone or combination treatment (coix seed extract plus gemcitabine; 10 mg/mL + 3 µg/mL, 5 mg/mL + 1.5 µg/mL, 2.5 mg/mL + 0.75 µg/mL, 1.25 mg/mL + 0.37 µg/mL, 0.63 mg/mL + 0.19 µg/mL and 0.063 mg/mL + 0.094 µg/mL), or DMSO for indicated time. At the end of each time point MTT assays were performed as reported previously [30]. Briefly, all cell culture medium was removed and replaced by fresh medium, then 20 µL MTT solution (5 mg/mL) was added to each well. The cells were incubated for another 4h at 37 °C in the dark. Formed formazan crystals were dissolved in 100 µL DMSO and the absorbance was measured at 570 nm on a microplate reader (Bio-Rad, Hercules, CA, USA). Tumor cell viability and IC_50_ were calculated. CI values and Fa value were calculated using CompuSyn software according to the instructions (ComboSyn, Inc., Paramus, NJ, USA). All assays were performed in triplicate.

### 4.4. Animal Study

BALB/c nude male mice (4 weeks old) were supplied by the Laboratory Animal Centre, Zhejiang Chinese Medical University (Zhejiang, China). The animal study was approved by the Animal Research Ethics Committee of Zhejiang Chinese Medical University (Pro#2014-03-018, 6 Mar 2014). All experiments were performed in accordance with the guidelines for the care and use of animals as established by Zhejiang Chinese Medical University (SYXK(H)2012-0002) and with respect to the European Communities Council Directive of September 22, 2010 (2010/63/EU).

BxPC3^luc^ cells were suspended in saline and diluted into a 1 × 10^7^ /mL cell suspension. 75% isopropyl alcohol was used to sterilize the left buttock skin of mice, and 0.2 mL of cell suspension was inoculated into the left side of nude mice near the posterior limb. Subcutaneous apophysis is a sign of a successful injection. When subcutaneous tumors developed to 100 mm^3^ in size, all the 4 week-old male mice (*n* = 24) were randomly divided into four groups: the coix seed extract group (12.5mL/kg, i.v. daily), the gemcitabine alone group (50mg/kg, i.v. 3/week), the coix seed extract and gemcitabine combination (coix seed extract 12.5 mL/kg, i.v. daily and gemcitabine 50 mg/kg, i.v. 3/week) and the control group (0.9% saline, i.v. daily). All mice were weighed daily after the first administration, and any symptoms, diet and drinking behaviors were observed. The average tumor volumes of each treatment group as well as solvent group were measured every three days during the whole drug treatment cycle. Tumor volume was calculated using the formula: V = (a×b^2^)/2, where “a” is the longest diameter, “b” is the shortest. The tumor volume and weight in nude mice were used to calculate the tumor inhibition rate in each experimental group [53]. Tumor tissues were fixed in buffered formalin for further analyses.

### 4.5. Luciferin Assay and Pharmacokinetic Analysis

#### 4.5.1. In Vitro Bioluminescent Assay

BxPC3^luc^ cells were seeded onto 96-well plates at a density of 8×10^3^ cells/well and incubated overnight. For single drug treatment, all cells were exposed to coix seed extract (10 mg/mL) or gemcitabine (3 μg/mL) for 24, 48, or 72 hours. For drug combination treatment, cells were exposed to coix seed extract (10 mg/mL) and gemcitabine (3 μg/mL) for 24, 48 or 72 hours. The same volume of DMSO was added to cells for those that did not receive combination therapy. At each time point, the solutions of each well were removed and washed twice with PBS. Then 100 μL of cell culture medium with 5 µg/mL of D-luciferin was added to each well and immediately imaged using a Xenogen IVIS kinetic system (Caliper Co. Ltd, Alameda, CA, USA). The photon intensity of each plate was detected and the signal was acquired every 2 min for 0 to 40 min to obtain the kinetics of D-luciferin. The bioluminescent intensity of each group was normalized by protein concentration and regarded as relative bioluminescent intensity (BLI_rel_). The protein concentration of the cells was determined by the BCA method as the manufacturer’s instructions. Each treatment point was measured at least six times within one assay. All assays were performed in triplicate.

#### 4.5.2. In Vivo Bioluminescent Assay

At days 7, 14 and 25 after the first administration of treatment, all mice were weighed and anesthetized by isoflurane gas and injected with 75 mg/kg of D-luciferin solution intravenously. Thereafter mice were placed in the Xenogen IVIS kinetic system (Caliper Co. Ltd, Alameda, CA, USA), and the bioluminescent signals of each mouse were recorded every 2 min within 140 min. The bioluminescent signals intensity of each mouse was normalized by tumor volume and considered as BLIrel. Each treatment point was measured at least six times within one assay. All assays were performed in triplicate.

#### 4.5.3. Bioluminescent Pharmacokinetic Parameters

The pharmacokinetic parameters AUC_luc_, K_luc_, and MRT_luc_ which represented the D-luciferin amounts in the pancreatic cancer cells were calculated using DAS 4.0 pharmacokinetic analysis software (Anhui, China), rendering the relational graph between BLI_rel_ and time as reported previously [17]. According to the noncompartment model, the kinetic parameters of BLI_rel_ such as the area under the curve (AUC_luc_), elimination rate constant (K_luc_), and mean resident time (MRT_luc_) were determined. The formulae for major parameters are as follows:

(1) $$AUC_{luc}=\int_{0}^{\infty }BLI_{rel}dt$$

(2) $$K_{\mathrm{luc}}=\frac{0.693}{t_{\frac{1}{2}}}$$

(3) $$MRT_{luc}=\int_{0}^{\infty }tBLI_{luc}dt/\int_{0}^{\infty }BLI_{luc}dt=\frac{AUMC_{luc}}{AUC_{luc}}$$

### 4.6. Immunohistochemical Analysis and Assessment

The immunohistochemistry assays were performed as reported previously [30]. The tumor sections were fixed in 4% paraformaldehyde for 24 hours, then embedded in paraffin. The sectioned (4-µm) tumor tissues were incubated with antibodies against Ki-67 (1:100), ABCB1 (1:200) and ABCG2 (1:200). The tissue was assessed for the protein expression in neoplastic areas. Integrated Optical Density (IOD) of Ki-67, ABCB1 and ABCG2 expression was recorded for each area (total or membrane). 

### 4.7. Western Blot Analysis

BxPC3^luc^ cells were exposed to selected drugs for 48 h, all cells were harvested and the protein level of ABCG2 and ABCB1 was evaluated. The membrane protein was extracted according to the manufacturer’s instructions for the ProteoJET Membrane Protein Extraction Kit (Fermentas, Burlington, Ontario, Canada). The cell lysate (50 µg) was subjected to electrophoresis on 4–12% sodium dodecyl sulfate (SDS)–polyacrylamide gels followed by transfer onto a polyvinylidene difluoride (PVDF) membrane using a glycine transfer buffer. After blocking in a blocking buffer containing 5% nonfat milk for 1 hour at room temperature, the membrane was incubated with anti-ABCG2 and anti-ABCB1 primary antibody (Santa Cruz Biotechnology Inc., Santa Cruz, CA, USA) overnight at 4 ℃, and incubated with goat anti-rabbit IRDye 800CW secondary antibody (LICOR Inc., Lincoln, NE, USA) for 2 hours at room temperature. The β-actin antibody and Na^+^/K^+^ ATPase α (H-3) were served as a loading control for total protein and membrane protein expression assessments respectively. Then the bands were visualized by Odyssey infrared imaging system (LICOR Inc., Lincoln, NE, USA).

### 4.8. Statistical Analysis

All data were calculated as mean ± standard deviation (SD) for at least three independent experiments. Statistical analysis of multiple group comparisons was performed by one-way analysis of variance (ANOVA). Comparisons between two groups were analyzed using Student’s t-tests. The association between pharmacokinetic parameters and PD_luc_ (TGI value *in vivo* and IC_50_ value *in vitro*) was analyzed by Pearson analysis [18,46]. A Pearson correlation analysis indicated a strong relationship between bioluminescent pharmacokinetics and pharmacodynamics in vitro and in vivo. The *in-vitro* and *in-vivo* correlation of kinetic parameters for different drugs treatment groups were analyzed by least square linear regression. A *p*-value less than 0.05 were considered to indicate a statistically significant result.

## Figures and Tables

**Table 1:** Pharmacokinetic parameters of the relative *in-vitro* bioluminescence imaging data.

Group	AUC_luc_(* 10^5^, BLI_rel_ × min)			MRT_luc_ (min)			K_luc_(* 10^−3^, min^−1^)		
	24 h	48 h	72 h	24 h	48 h	72 h	24 h	48 h	72 h
DMSO	3136.00 ± 620.56	1201.00 ± 218.90	802.00 ± 54.63	17.20 ± 0.60	18.96 ± 0.40	19.20 ± 0.60	7.80 ± 1.00	8.10 ± 0.39	8.73 ± 0.40
GEM	1853.00 ± 273.86 ^**^	327.00 ± 63.86 ^**^	319.00 ± 63.91 ^**^	17.18 ± 0.60	17.30 ± 0.60 ^**^	16.31 ± 0.20 ^**^	9.40 ± 1.90	14.48 ± 2.09 ^**^	20.03 ± 3.18 ^**^
CSE	5076.00 ± 164.66	1974.00 ± 9.20	1220.00 ± 173.40	20.31 ± 0.09	20.92 ± 0.20	19.54 ± 0.50	7.09 ± 0.49	8.10 ± 0.50	11.03 ± 1.27
GEM + CSE	2334.50 ± 182.18 ^##^	524.17 ± 127.43 ^##^	533.00 ± 82.27 ^##^	18.64 ± 0.62 ^##^	17.64 ± 0.50	17.06 ± 0.50 ^##^	9.44 ± 1.50 ^##^	12.56 ± 0.59 ^##^	16.06 ± 0.62 ^##^

*n* = 6, mean ± SD. ** *p* < 0.01 vs. DMSO; ## *p* < 0.01 vs. GEM Group. The bioluminescent intensity of each group was normalized by protein concentration and regarded as relative bioluminescent intensity (BLI_rel_). GEM, gemcitabine (3 µg/mL); CSE, coix seed extract (10 mg/mL); GEM + CSE, gemcitabine and coix seed extract combination treatment (3 µg/mL + 10 mg/mL); DMSO, dimethyl sulfoxide; AUC, area under the curve; MRT, mean resident time; K, elimination rate constant; BLI_rel_, relative bioluminescence imaging.

**Table 2:** Pharmacokinetic parameters of the relative *in-vivo* bioluminescence imaging data.

Group	AUC_luc_(* 10^7^, BLI_rel_ × min)			MRT_luc_ (min)			K_luc_(* 10^−3^, min^−1^)		
	7d	14d	25d	7d	14d	25d	7d	14d	25d
Solvent	1074.67 ± 164.83	1095.83 ± 61.65	1013.33 ± 67.00	22.69 ± 1.13	23.20 ± 1.42	24.40 ± 2.10	16.17 ± 5.38	22.65 ± 2.42	24.28 ± 3.41
GEM	250.67 ± 138.70 ^**^	229.00 ± 23.19 ^**^	182.00 ± 94.16 ^**^	18.70 ± 1.35 ^**^	16.89 ± 2.89 ^**^	16.13 ± 0.66 ^**^	26.03 ± 1.63 ^**^	29.07 ± 0.76 ^**^	34.97 ± 3.20 ^**^
CSE	2321.83 ± 65.00	2366.67 ± 177.91	2554.00 ± 134.65	34.05 ± 0.41	37.23 ± 3.02	44.37 ± 1.86	12.03 ± 1.35	14.00 ± 3.89	15.97 ± 1.85
GEM + CSE	718.00 ± 75.83 ^##^	796.17 ± 269.14 ^##^	658.83 ± 132.24 ^##^	20.53 ± 0.34 ^##^	23.36 ± 0.68 ^##^	26.21 ± 2.81 ^##^	15.03 ± 1.49 ^##^	23.03 ± 1.01 ^##^	23.03 ± 1.83 ^##^

*n* = 6, mean ± SD. ** *p* < 0.01 vs. DMSO; ## *p* < 0.01 vs. GEM Group. The bioluminescent intensity of each group was normalized by protein concentration and regarded as relative bioluminescent intensity (BLI_rel_). GEM, gemcitabine (3 µg/mL); CSE, coix seed extract (10 mg/mL); GEM + CSE, gemcitabine and coix seed extract combination treatment (3 µg/mL + 10 mg/mL); DMSO, dimethyl sulfoxide; AUC, area under the curve; MRT, mean resident time; K, elimination rate constant; BLI_rel_, relative bioluminescence imaging.

**Table 3:** The *in-vitro* and *in-vivo* association of bioluminescent pharmacokinetic parameters.

Group	Pearson Coefficient	*p* Value
GEM + CSE		
AUC_luc_ *in vitro v.s*	0.723	< 0.001
AUC_luc_ *in vivo*		
MRT_luc_ *in vitro v.s*	−0.752	< 0.001
MRT_luc_ *in vivo*		
K_luc_ *in vitro* v.s	0.735	< 0.001
K_luc_ *in vivo*		
GEM		
AUC_luc_ *in vitro v.s*	0.762	< 0.001
AUC_luc_ *in vivo*		
MRT_luc_ *in vitro v.s*	−0.102	0.344
MRT_luc_ *in vivo*		
K_luc_ *in vitro* v.s	0.907	< 0.001
K_luc_ *in vivo*		
CSE		
AUC_luc_ *in vitro v.s*	0.994	< 0.001
AUC_luc_ *in vivo*		
MRT_luc_ *in vitro v.s*	−0.524	0.013
MRT_luc_ *in vivo*		
K_luc_ *in vitro* v.s K_luc_ *in vivo*	0.839	< 0.001

The pharmacokinetic parameters AUC_luc_, MRT_luc_ and K_luc_ are determined by using DAS 4.0 pharmacokinetic analysis software (Anhui, China) according to the noncompartment model. GEM, gemcitabine; CSE, coix seed extract; GEM + CSE, gemcitabine and coix seed extract combination treatment; AUC, area under the curve; MRT, mean resident time; K, elimination rate constant.

**Table 4:** The association between IC_50_ value/TGI value and the pharmacokinetic parameters by different treatment groups.

Group		Pearson Coefficient	*p* Value
***In Vitro* Association**			
IC_50_ value of GEM + CSE v.s.			
	AUC_luc_	−0.460	0.027
	MRT_luc_	−0.103	0.342
	K_luc_	0.436	0.035
IC_50_ value of GEM v.s.			
	AUC_luc_	−0.580	0.006
	MRT_luc_	−0.038	0.441
	K_luc_	0.422	0.041
IC_50_ value of CSE v.s.			
	AUC_luc_	−0.609	0.004
	MRT_luc_	−0.639	0.002
	K_luc_	0.403	0.049
*In vivo* association			
TGI value of GEM + CSE v.s.			
	AUC_luc_	0.391	0.054
	MRT_luc_	0.056	0.413
	K_luc_	−0.360	0.071
TGI value of GEM v.s.			
	AUC_luc_	0.452	0.030
	MRT_luc_	0.092	0.358
	K_luc_	−0.173	0.246
TGI value of CSE v.s.			
	AUC_luc_	0.334	0.087
	MRT_luc_	0.241	0.167
	K_luc_	−0.013	0.480

The pharmacokinetic parameters AUC_luc_, MRT_luc_ and K_luc_ are determined by using DAS 4.0 pharmacokinetic analysis software (Anhui, China) according to the noncompartment model. IC_50_ value and TGI value are used to evaluate the antitumor efficacy by different treatment groups *in vitro* and *in vivo*. GEM, gemcitabine; CSE, coix seed extract; GEM + CSE, gemcitabine and coix seed extract combination treatment; AUC, area under the curve; MRT, mean resident time; K, elimination rate constant; IC_50_, half maximal inhibitory concentration; TGI, tumor growth inhibition rate.

## Supplementary Materials

Supplementary materials can be found at https://www.mdpi.com/1422-0067/20/21/5250/s1.

## Abbreviations

ABC transportersAUC	ATP-binding cassette transportersarea under the curve
BLI	bioluminescence imaging
BLI_rel_	relative bioluminescence imaging
CSE	coix seed extract
DMSO	dimethyl sulfoxide
FBS	fetal bovine serum
GEM	gemcitabine
K	elimination rate constant
MDR	multidrug resistance
MRT	mean resident time
MTT	3-(4,5-Dimethylthiazol-2-yl)-2,5-diphenyltetrazolium bromide
PD	pharmacodynamics
PK	pharmacokinetics
SDS-PAGE electrophoresis	sodium dodecyl sulfate polyacrylamide gel electrophoresis
SNR	signal-to-noise ratio
